# Is the comprehension of idiomatic sentences indeed impaired in paranoid Schizophrenia? A window into semantic processing deficits

**DOI:** 10.3389/fnhum.2014.00799

**Published:** 2014-10-09

**Authors:** Francesca Pesciarelli, Tania Gamberoni, Fabio Ferlazzo, Leo Lo Russo, Francesca Pedrazzi, Ermanno Melati, Cristina Cacciari

**Affiliations:** ^1^Department of Biomedical, Metabolic, and Neurological Sciences, University of ModenaModena, Italy; ^2^Centro Salute Mentale PavulloModena, Italy; ^3^Villa Igea Private HospitalModena, Italy; ^4^Department of Psychology, Sapienza University of RomeRome, Italy; ^5^Centro Salute Mentale Polo Ovest ModenaModena, Italy

**Keywords:** paranoid schizophrenia, language comprehension, idioms, predictability, multiword units

## Abstract

Schizophrenia patients have been reported to be more impaired in comprehending non-literal than literal language since early studies on proverbs. Preference for literal rather than figurative interpretations continues to be documented. The main aim of this study was to establish whether patients are indeed able to use combinatorial semantic processing to comprehend literal sentences and both combinatorial analysis, and retrieval of pre-stored meanings to comprehend idiomatic sentences. The study employed a sentence continuation task in which subjects were asked to decide whether a target word was a sensible continuation of a previous sentence fragment to investigate idiomatic and literal sentence comprehension in patients with paranoid schizophrenia. Patients and healthy controls were faster in accepting sensible continuations than in rejecting non-sensible ones in both literal and idiomatic sentences. Patients were as accurate as controls in comprehending literal and idiomatic sentences, but they were overall slower than controls in all conditions. Once the contribution of cognitive covariates was partialled out, the response times (RTs) to sensible idiomatic continuations of patients did not significantly differ from those of controls. This suggests that the state of residual schizophrenia did not contribute to slower processing of sensible idioms above and beyond the cognitive deficits that are typically associated with schizophrenia.

## Introduction

Schizophrenia (SZ) is a chronic, debilitating illness characterized by perturbations in cognition, affect, and behavior (DSM-V; American Psychiatric Association, 2013). Most SZ patients have substantial cognitive impairments, compared to overall normative standards and to premorbid functioning, often including language, together with executive function, memory, and attention (for overviews, see Kuperberg and Heckers, 2000; Gold et al., 2009; Harvey, 2010; Barch and Ceaser, 2012; Fisher et al., 2013). SZ has been associated with widespread abnormality of a network of brain areas (e.g., a reversed laterality of activation in the superior temporal gyrus, morphological asymmetries in the superior temporal lobe, structural abnormalities of the ventral parts of the prefrontal cortex) that include the frontal and temporal cortex, the hippocampus, and subcortical regions (for overviews, see Kuperberg and Heckers, 2000; Mitchell and Crow, 2005). The brain areas with abnormal activation or morphology partially overlap with the areas necessary for language comprehension, and specifically for non-literal language comprehension (for overviews, see Thoma and Daum, 2006; Romero Lauro et al., 2008; Cacciari and Papagno, 2012). This brain dysfunction in SZ has been thought to underlie the clinical symptom of concretism (i.e., difficulty in interpreting abstract, non-literal language) that leads to impaired comprehension of non-literal complex structures (Kircher et al., 2007; Schettino et al., 2010; Mashal et al., 2013).

A vast literature on SZ patients has documented semantic processing impairments at single word and sentence levels (for overviews, see Condray et al., 2002; Kiang and Kutas, 2005; Pomarol-Clotet et al., 2008; Kuperberg, 2010a,b). At a word level, a wealth of behavioral and EEG studies compared semantic priming^1^ effects in SZ patients and healthy controls obtaining divergent results (for overviews, see Minzenberg et al., 2002; Pomarol-Clotet et al., 2008; Kuperberg, 2010a,b; Mathalon et al., 2010; Wang et al., 2011). Studies found an association between SZ and increased spread of activation to weak associates instead of, or in addition to, strong associates at short SOA (stimulus onset asynchrony, SOA: interval between the onset of prime and target presentations) (less than 300 ms). This hyper-priming effect was often accompanied by reduced or absent priming at long SOAs (more than 300 ms). The exact interpretation of different semantic priming effects at short and long lags is still disputed. For instance, according to the *Activation-Maintenance model* (Salisbury, 2004, 2008) disinhibition within semantic memory leads to the initial large automatic spread of activation in the mental lexicon that would be responsible for the hyper- priming effect often found at short SOAs. Activation would then decay as a function of bottom-up semantic memory trace dissipation (Neely, 1991) coupled with impairment in long-term top-down verbal working memory maintenance. Deficits in maintenance and use of contextual information would lead to impaired semantic priming at long SOAs. In sum, semantic dysfunction in schizophrenia would result from automatic over-activation in semantic networks at short lags and dysfunction in late, controlled processes of context use at long lags (Niznikiewicz et al., 2010). In fact, insensitivity to contextual information is thought to be one of the hallmarks of the linguistic behavior of SZ patients (e.g., Niznikiewicz et al., 1997; Kuperberg et al., 1998; Cohen et al., 1999; Titone et al., 2002). Failure in using contextual information may reflect a more general inability of patients to construct and maintain an internal representation of context for control of action (Cohen and Servan-Schreiber, 1992). This has been correlated with deficits in maintaining context in working memory (e.g., Cohen et al., 1999; Barch et al., 1996). Patients may fail to efficiently use contextual information also because of their inability to identify and encode contextually relevant information (Chapman et al., 1976). However, Titone et al. (2000, 2002) documented that, under specific circumstances, SZ patients may activate contextually relevant information but may fail in inhibiting contextually-irrelevant information especially at long SOAs (Minzenberg et al., 2002) because of a general deficit in controlled semantic processing.

At a sentence level, processing deficits in SZ patients appeared in different forms including syntactic, semantic and pragmatic aspects (e.g., Kuperberg et al., 1998, 2006; Ditman and Kuperberg, 2007, 2010). For instance, it has been shown that SZ patients are relatively insensitive to semantic anomalies presumably because of impairment in building up context during online language processing (Ditman and Kuperberg, 2007). At least some of the sentence-level comprehension abnormalities observed in SZ patients were thought to arise (Kuperberg, 2007, 2010b) from *an imbalance in activity between semantic-memory based and combinatorial mechanisms: unlike healthy controls, patients may fail to engage in combinatorial processing; interpretation (and possibly production) may therefore be primarily driven by semantic memory-based processes* (Kuperberg, 2010b, p. 597). In sum, in SZ these two streams of analysis would fail to cooperate and interact to produce the final sentence interpretation while in normal comprehenders the semantic memory-based stream of analysis occurs partly in parallel with the combinatorial stream of analysis in which the lexico-semantic information of individual words is integrated compositionally with morphosyntactic and thematic structures to determine the sentence meaning.

In normal language comprehension, the general function of combinatorial semantic processing is to integrate the meaning of single words into a coherent sentence representation. However, language comprises many different materials whose actual comprehension requires going beyond compositional processes. In fact for comprehending multiword units such as, for instance, idioms (e.g., *break the ice*, *beat about the bush*), binomials (e.g., *bride and groom*, *spic*, *and span*), or collocations (e.g., *black coffee, morning sickness*), it is necessary to merge combinatorial single word processing with retrieval of lexicalized meanings (for overviews, see Siyanova-Chanturia, 2013; Cacciari, 2014). Establishing whether SZ patients are indeed able to use combinatorial semantic processing in literal sentence comprehension and both combinatorial analysis and retrieval of stored, global meanings in idiomatic sentence comprehension is the main aim of this study.

## Deficits in the comprehension of non-literal language in SZ

SZ patients have been reported to be more impaired in comprehending non-literal than literal language since early studies on proverbs and metaphors (Gorham, 1961; Kasanin, 1994). Impairment in the comprehension of non-literal language continues to be documented in terms of preference for literal rather than figurative interpretations and poor appreciation of irony (*literality bias*) (metaphors: Chapman, 1960; Cutting and Murphy, 1990; Spitzer, 1997; Drury et al., 1998; Langdon et al., 2002; Langdon and Coltheart, 2004; Kircher et al., 2007; Mashal et al., 2013; idioms: Titone et al., 2002; Iakimova et al., 2005, 2006, 2010; Schettino et al., 2010; proverbs: Gorham, 1961; de Bonis et al., 1997; Sponheim et al., 2003; Brüne and Bodenstein, 2005; Kiang et al., 2007; Thoma et al., 2009; irony: Herold et al., 2002; Langdon et al., 2002; Rapp et al., 2013).

Poor understanding of non-literal language has been attributed to a variety of factors, including a generalized pragmatic comprehension deficit (Tavano et al., 2008). However, recently the idea of a unique mechanism underlying non-literal language deficits has been questioned (Martin and McDonald, 2003; Champagne-Lavau et al., 2006, 2007) by studies that observed qualitatively distinct deficits in different types of non-literal expression, notably in metaphor comprehension (Iakimova et al., 2005; Elvevåg et al., 2011), appreciation of irony (Langdon et al., 2002) and idioms (with poorer performances on literally plausible than on literally implausible idioms, e.g., *skate on thin ice* vs. *throw caution to the winds*) (Titone et al., 2002; Iakimova et al., 2010; Schettino et al., 2010). Then deficient comprehension of non-literal language has been attributed to poor theory of mind (ToM), defined as the ability to attribute mental states to oneself and the others in order to explain and predict behavior in social contexts (Brüne, 2005; Brüne and Bodenstein, 2005; Mo et al., 2008; Champagne-Lavau and Stip, 2009; Gavilán and García-Albea, 2010; Schettino et al., 2010; but see Langdon et al., 2002; Varga et al., 2014). Impaired figurative language comprehension has also been linked to inadequate use of contextual information to construct abstract figurative meanings (Strandburg et al., 1997; Kircher et al., 2007). However, Titone et al. (2002; see also Iakimova et al., 2006, 2010) questioned the idea that SZ patients necessarily exhibit a literality bias. In fact, the lexical decision study of Titone et al. showed that SZ patients were as able as control subjects to use idiomatic contexts to generate idiomatic interpretations when the idiomatic meaning was literally implausible (e.g., *come up roses*) but they instead failed when the idiomatic meaning was semantically ambiguous having also a literal counterpart (e.g., *break the ice*). In sum, this selectively spared ability to comprehend unambiguous idioms would confirm that patients do not have difficulty in understanding non-literal meanings *per se*. Rather they would fail in suppressing competing literal meanings: *difficulty in inhibiting literal interpretation of idiomatic phrases when one is possible, and/or processing ambiguous stimuli, are the sources of contextual failures in schizophrenia* (Titone et al., 2002, p. 318). Unfortunately, in Titone et al's study (2002) patients were presented only with idiomatic sentences, hence without any literal sentence control condition. Hence it is impossible to establish whether patients were comparably good at incrementally integrating word meanings in a compositional way (as necessary for literal sentence comprehension) and at retrieving prefabricated idiomatic meanings from semantic memory (as necessary for idiomatic sentence comprehension). Differences between the comprehension of literally plausible and implausible idioms were observed also by Schettino et al. (2010) in a picture-sentence matching task study. SZ patients and healthy controls were presented with literal and idiomatic sentences followed by a picture correctly or incorrectly depicting the sentence meaning. SZ patients were impaired in choosing the appropriate picture in both types of idiomatic sentence, with a particularly poor performance for literally plausible idioms. However, this result may be influenced by the difficult of representing idiomatic abstract meanings in a pictorial format. Literal pictures may have been easier to elaborate than idiomatic pictures leading to underestimation of the actual ability of patients to comprehend idioms (Papagno and Caporali, 2007).

## The present study

The present study aimed at investigating whether SZ patients were indeed able to use combinatorial semantic processing to comprehend literal sentences and both combinatorial analysis and retrieval of pre-stored meanings to comprehend idiomatic sentences. In fact, idioms are strings of words with a highly conventionalized meaning stored in long-term semantic memory. Idiomatic meaning does not derive from the composition of idiom constituent word meanings and often refers to abstract mental states or events. We used an online sentence continuation verification task and controlled for a factor that is known to play a major role in literal and non-literal language comprehension, namely the predictability of incoming words. In the sentence continuation verification task, participants are asked to decide whether a target word is a sensible continuation of a previous sentence fragment. This relatively easy task has been widely employed in the psycholinguistic literature to assess sentence comprehension (Burgess and Shallice, 1996) since it is well suited to obtain information on moment-by-moment comprehension placing at the same time little demand on the need to maintain and update information in working memory. The presentation of both the sentence fragment and the target word were self-paced rather than being regulated at fixed rates because self-paced methods are known to allow subjects to read at a pace that matches their internal comprehension processes (Just and Carpenter, 1980; Kuperberg et al., 2006). Using similar, fixed time durations for patients and controls would have been problematic also because evidence (Butler et al., 2002; Quelen et al., 2005) showed that typically SZ patients need longer presentation durations to perceive a stimulus.

We used only idioms without a literal counterpart (i.e., literally implausible strings, see the Appendix in Supplementary Material and **Table 2** for examples), because evidence showed that SZ patients may be deficient in strategically using contextual information for inhibiting competing literal interpretations when idioms also possess a literal meaning (Titone et al., 2002; Schettino et al., 2010). Since idioms typically have a prefabricated structure, their presence in a sentence may be determined in advance, or reasonably predicted, based on part of an idiom string (e.g., *carry the world on one's…* triggers high expectations for the idiomatic completion *shoulder*) (Cacciari and Tabossi, 1988). Several behavioral and EEG studies on language-preserved participants showed that predictable idioms are understood faster than unpredictable ones (e.g., Cacciari and Tabossi, 1988; Cacciari et al., 2007; Vespignani et al., 2010); when the initial fragment of a string creates high expectancy about a final idiomatic conclusion, recognition of a word providing an unexpected ending is slowed down (Tabossi et al., 2005). In sum, idiom predictability can constrain the search through semantic memory facilitating the processing of anticipated components or hindering that of unpredicted ones. However, notwithstanding the acknowledged relevance of word predictability in language processing (for overviews see Federmeier, 2007; Davenport and Coulson, 2011; Cacciari, 2014), this factor has been rather neglected in previous idiom studies on SZ patients^2^. Hence we manipulated the predictability of sentence-final words designing literal and idiomatic sentences whose final words were comparably highly expected. While we expect healthy controls to be equally facilitated in anticipating what comes next in literal and idiomatic sentences, patients may be more facilitated by idiomatic than literal predictability because of the bound pre-fabricated structure of idioms.

As we mentioned, in her *Dual Stream hypothesis* Kuperberg (2007, 2010a,b) argued that SZ patients may be characterized by overreliance on semantic-memory based stream of language processing at the expenses of the combinatorial processing stream. Paradoxically, overreliance on semantic memory-based language processing may turn out to be more detrimental to literal than to idiomatic language comprehension. In fact, if one assumes that idiomatic meanings do not have to be compositionally established but are directly retrieved from semantic memory, then this would imply that idiom interpretation in SZ patients should be even more reliant on semantic-memory based processes than in healthy controls. In contrast, comprehending literal sentences requires syntactic and semantic integration of the constituent word meanings. Hence SZ patients may perform nearly as well as healthy controls in comprehending idiomatic ready-to-go meanings, when idioms did not have a competing literal counterpart, while being impaired in understanding literal sentences, at variance with the *literality bias* suggested by prior studies. Kuperberg (2010b) argued that retrieval of idioms with a literal counterpart (i.e., ambiguous idioms such as, for instance, *break the ice*) could be relatively facilitated because *a relative impairment in engaging additional combinatorial processing to construct the implausible literal meaning of such idioms* [may result] *in less conflict and increased access to the stored idiomatic meaning* (p. 596). Here we argue that this may also be true of idioms without a literal counterpart (as those used in this study) reflecting a general imbalance of SZ patients toward semantic memory-based processing.

The literature indicates that SZ patients tend to be slower than healthy controls on most cognitive measures (Vinogradov et al., 1998; Harvey, 2010). This may artificially increase the reaction time difference between groups. Hence finding slower response times (RTs) in patients than in healthy controls may not be sufficient for concluding that comprehension is impaired. To overcome this problem, often semantic priming studies (e.g., Spitzer et al., 1993; Kiefer et al., 2009) analyzed the effect of prior context on target word in terms of a priming score (PRI) (see Methods Section). PRIs would reflect the amount of facilitation of prior context on the RTs to a target word (Spitzer et al., 1993). Although the use of PRI primarily derives from single word semantic priming studies, we measured the PRIs of patients and healthy participants when sentence-final words completed literal and idiomatical sentences in sensible or non-sensible ways assuming that sentence-final words could be facilitated by the previous sentence fragments. As reported in the Introduction, in SZ deficient semantic processing may produce distorted priming effect at short lags such that access to words preceded by related primes may be abnormally increased (or reduced) (Ditman and Kuperberg, 2007). Hence, patients, unlike controls, may exhibit exaggerated contextual priming on correct target words as reflected by PRIs larger in patients than in controls.

Studies documented that abnormal semantic processing is often closely associated with evidence of thought disorders, especially in severely ill patients (Ditman et al., 2011). This multidimensional disturbance may emerge in both language comprehension and production with loose lexical associations, incoherent language production, deficient abstract thinking and semantic memory deficits (Andreasen, 1979; Kuperberg and Heckers, 2000; Pomarol-Clotet et al., 2008; Salisbury, 2008; Levy et al., 2010). These disorders are thought to be particularly detrimental to non-literal language comprehension (Iakimova et al., 2010; Schettino et al., 2010; Mashal et al., 2013). Although the severity of the clinical profiles of the SZ patients involved in this study went from mild to moderate, we tested possible effects of thought disorder (as reflected by scores in the *Positive and Negative Syndrome Scale*, *PANSS*) on target word processing.

We tested a group of relatively young patients (20–45 years-old) characterized by mild-to-moderate forms of paranoid SZ (as reflected by PANSS scores) and ongoing clinical stability. The choice of this clinical profile was motivated by evidence that in general paranoid SZ patients (together with schizoaffective patients) have higher levels of cognitive ability relative to other forms of the disorder (Goldstein et al., 2005). This may result in a patient sample with relatively moderate average level of psychopathology limiting the potential of any inference about illness state effects on language comprehension but with the advantage of possibly showing aberrant language comprehension already in mild-to-moderate forms of this complex pathology.

In summary, the general aim of the study was to test whether overreliance on the semantic-memory based stream of language processing, at the expenses of the combinatorial processing stream, may paradoxically lead to less impaired comprehension of idiomatic than of literal sentences. SZ patients, unlike healthy participants, may in fact perform worse on literal sentences that require full combinatorial analysis than on idiomatic meanings that do not have to be compositionally established but are directly retrieved from semantic memory. However, SZ impaired language processing may produce distorted semantic effect such that patients, unlike controls, may exhibit exaggerated contextual priming effects. Lastly, we expect the severity of thought disorders within the patient group to affect both RTs and accuracy.

## Experiment

### Methods

#### Participants

Participants consisted of 39 (14 female; mean age = 31 years, age range = 20–45, *SD* = 6.2) chronic outpatients with paranoid SZ (DSM-V; American Psychiatric Association, 2013) and 39 healthy volunteers as control participants. Italian was the native language of all participants. The general inclusion criteria were at least 10 years of formal education and age between 18 and 45 years. Patients were recruited from the geographically defined catchment area of Modena and treated by the West Modena Mental Health Service and by a clinic reporting to the same Mental Health Daycare district. Healthy control participants were volunteers recruited in the community through public advertisements. Controls were pairwise matched to patients for age (±2), sex, and education (±2) (see Table 1). Controls self-reported to have no history of alcohol or substance abuse, no major medical or neurological illness and no psychiatric illness in first degree relatives. To exclude any past or present psychiatric disorder, controls were administered the *Brief Psychiatric Rating Scale* (BPRS, Ventura et al., 1993). The diagnosis of paranoid schizophrenia of patients was based on the *Positive and Negative Syndrome Scale* (PANSS; Kay et al., 1987; score = 46.69, range = 34–68, *SD* = 8.1) and it was confirmed by the clinical consensus of staff psychiatrists. The PANSS is a semi structured interview designed to assess the presence and severity of positive (7 items, e.g., *hallucinations*, *conceptual disorganization*), negative (7 items, e.g., *emotional withdrawal*, *difficulty in abstract thinking or concretism*), and general (16 items, e.g., *anxiety*, *unusual thought content*) psychopathological symptoms. The interview was administered to patients by senior psychiatrists blind to the cognitive data and was aimed at assessing the patients' symptom status in the past week. Based on PANSS classification criteria, 35 patients had a mild form of SZ (PANSS Total score from 34 to 55) and four a moderate form (from 61 to 68)^3^. At time of testing, all patients were responsive and clinically stabilized. None of them had comorbid psychiatric disorders, alcohol, or substance abuse prior to the study, history of traumatic head injury with loss of consciousness, epilepsy, or other neurological diseases. 33 of the 39 patients were prescribed second-generation antipsychotic medications (as defined by Lohr and Braff, 2003), two first-generation antipsychotics, and four a combination of first- and second-generation antipsychotics. At time of testing, patients had a mean IQ of 88 (range = 58–126, *SD* = 18), assessed with the *Wechsler Adult Intelligence Scale* (WAIS-R), a mean education of 12.6 years (range = 10–14, *SD* = 1.33), and a mean illness duration of 8.97 years (range = 1–29, *SD* = 5.94) (see Table 1). A set of neuropsychological tests was administered to patients and control participants to assess general cognitive functions and language (Table 1). Specifically, both patients and controls were administered the Syntactic competence sub-scale of the *Batteria per l'analisi dei deficit afasici* (*B.A.D.A.*, Miceli et al., 1994), an Italian battery on language comprehension originally designed for aphasic patients, to assess basic syntactic comprehension ability and the *Phonemic and Semantic Fluency Tests* (Italian Version; Novelli et al., 1986) to assess general cognitive functioning and semantic processing deficits (for overviews, see Henry and Crawford, 2005). In the *Phonemic fluency test*, individuals produce as many words beginning with given letters (in Italian, F, P, L) as possible in a time interval (60″ for each letter). In the *Semantic fluency test*, individuals produce as many members of given stimulus categories (car brands, fruits, and animals) as possible in a time interval (60″ for each category). For controls, Digit Span and Vocabulary subtests of WAIS-R were used to estimate, respectively, verbal short-term memory and global verbal intelligence function (Lezak et al., 2004). Patients had significantly poorer performances than healthy controls in all tests (Table 1).

**Table 1:** **Demographic characteristics of the study sample, and clinical characteristics of the schizophrenic patients**.

	**Patients**				**Controls**				
	**Mean**	**Min**.	**Max**.	***SD***	**Mean**	**Min**.	**Max**.	***SD***	***p***
Sex	M = 25; F = 14				M = 25; F = 14				
Age (years)	31.41	20	45	6.22	31.28	19	45	6.31	0.93
Education (years)	12.56	10	17	1.33	12.51	10	17	1.48	0.88
Drug	SG = 33; FG = 2; FSG = 4								
Years of illness	8.97	1	29	5.94					
WAIS-R (verbal scale)	91.05	62	118	15.41					
WAIS-R (performance scale)	86.31	58	121	19.42					
WAIS-R (total score)	87.82	58	126	18.31					
Vocabulary (WAIS-R)	8.23	3	15	3.24	10.77	7	17	2.38	0.0001
Phonemic fluency	28.51	15	54	8.25	37.28	23	58	7.68	0.0001
Semantic fluency	38.44	25	62	8.44	44.10	23	56	7.74	0.003
BADA (errors)	1.15	0	5	1.18	0.03	0	1	0.16	0.0001
Digit SPAN (forward)	5.44	3.5	7.5	0.74	5.85	4.5	7.75	0.83	0.04
Digit SPAN (backward)	3.75	1.69	6.42	1.07	4.28	1.47	6.47	0.97	0.05
Digit SPAN (total score)	9.18	6.44	13.29	1.51	10.13	6.97	13.92	1.57	0.02
BPRS					2	2	2	0	
PANSS (positive scale)	11.64	7	19	3.12					
PANSS (negative scale)	11.21	7	26	4.02					
PANSS (general psychopathology scale)	23.84	18	34	3.43					
PANSS (total score)	46.69	34	68	8.13					

M, male; F, female; FG, first-generation antipsychotics; SG, second-generation antipsychotics; FSG, combination of first- and second–generation antipsychotics.

Written informed consent was obtained from all participants. Permission for the study was obtained from the Ethical Committee of Modena (*Comitato Etico Provinciale, Azienda Ospedaliero-Universitaria di Modena*).

#### Materials

Experimental stimuli were formed by 38 idiomatic and 38 literal sentences (see Table 2 for examples, and the Appendix in Supplementary Material for the idiom list). The final words of all sentences were highly predictable in context, as shown by cloze probability values (see below). Prior to the study, we performed several tests to norm the experimental materials on language-unimpaired subjects (not involved in any other phases of the experiment). First, 60 idioms without a plausible literal meaning were selected from an Italian Idiom Dictionary (e.g., *avere dei grilli per la testa*, *to be full of strange ideas*, *mettersi il cuore in pace, to put one's mind at rest*) and were divided into two lists. Each list was submitted to 20 participants who rated the familiarity of each idiom (from 1: *Never heard* to 7: *Heard very often*) and provided a meaning paraphrase. The 38 idioms selected as experimental materials were highly familiar (*M* = 5.02, *SD* = 0.59, range = 3.69–5.94) and were correctly paraphrased (*M* = 88.8%, *SD* = 8.2, range = 76–100%). Idioms were formed on average by 5.3 words (*SD* = 0.7, range = 4–7). Then, 38 sentences (mean number of words = 7.5, *SD* = 1.01, range = 6–10) ending with the idiom string and without any bias to the idiomatic meaning were created together with 38 literal sentences of comparable length and syntactic structure (mean number of words = 7.7, *SD* = 1.02, range = 6–10; *t* < 1) (see Table 2 for examples). To test the cloze-probability of sentence-final words (i.e., the probability that a specific word is given to complete a specific sentence context), different questionnaires containing sentence fragments of increasing length were created. 90 different healthy participants were asked to complete the sentence fragments with the first word that came to their mind. In the final set of experimental materials, idiomatic and literal final words had statistically indistinguishable, very high cloze probability mean values (*M* = 0.90; *SD* = 0.8, range = 0.75–1; Idiomatic sentences: *M* = 0.89, *SD* = 0.7, range = 0.75–1; Literal sentences: 0.91, *SD* = 1.4, range = 0.76–1, *t* < 1).

**Table 2:** **Examples of experimental sentences in Italian and with word-by-word English translations**.

	**Sensible**	**Non-sensible**	
**IDIOMATIC SENTENCES**			
Giulia aveva dei grilli per la (Giulia had some crickets for the)	TESTA (HEAD)	SPUGNA (SPONGE)	Giulia was full of strange idea
Ilenia faceva di ogni erba un (Ilenia made of each herb a)	FASCIO (BUNDLE)	TRAVE (BEAM)	Ilenia lumped everything together
Carlo si mise il cuore in (Carlo put the heart in)	PACE (PEACE)	BASE (BASE)	Carlo resigned himself to it
Pino si sentiva in una botte di (Pino felt himself in a barrel of)	FERRO (IRON)	GUANTO (GLOVE)	Pino felt very sure
**LITERAL SENTENCES**			
Maria alla sera andava a nuotare in (Maria at night went swimming at the)	PISCINA (POOL)	CRATERE (CRATER)	
Roberto cadde e si fece molto (Roberto felt down and made himself a lot of)	MALE (ACHE)	CALDO (HOT)	
Simona si asciugò i capelli con il (Simona dried her hair with the)	PHON (AIRDRYER)	SEME (SEED)	
Giorgio allentò la cravatta intorno al (Giorgio loosened the tie around the)	COLLO (NECK)	BRODO (BROTH)	

Good and bad continuations are indicated in capital letters. The idiom meaning is provided in parentheses.

The 38 literal and 38 idiomatic sentences were presented in two conditions. In the Sensible continuation Condition, the sentence-final word was the word that obtained the highest cloze value in the norming phase. These corresponded to the idiom-final words in idiomatic sentences. In the Non-Sensible condition, the last words of idiomatic and literal sentences were substituted with unexpected constituents (cloze value equal zero in both conditions), semantically incongruent to the idiomatic or literal meaning of the sentence, and without any association to any of the preceding words (e.g., idiomatic sentence: *Giulia aveva dei grilli per la TESTA/SPUGNA*, *Giulia had some crickets for the HEAD/SPONGE, Giulia was full of strange idea*; literal sentence: *Maria alla sera andava a nuotare in PISCINA/CRATERE, Maria at night went swimming in the POOL/CRATER*). In order to ensure that the effects of interest were not linked to specific word characteristics, the words forming sensible and non-sensible continuations in each condition were matched for grammatical class, length, frequency, and Age of Acquisition (AoA). In addition, we included 76 filler sentences without any idiom strings whose last word had low to medium cloze probability. The last constituent completed the sentences in sensible ways in half filler sentences and in non-sensible ways in the remaining half. Two lists were created and participants were randomly assigned to one of the two lists so that each sentence was presented only in the sensible or non-sensible version. Each list contained 152 sentences: 38 sentences with sensible continuations (19 idiomatic and 19 literal), 38 sentences with non-sensible continuations (19 idiomatic and 19 literal), and 76 filler literal sentences (38 sensible and 38 non-sensible). Idiomatic sentences represented only 25% of the total number of sentences to prevent participants from developing specific processing strategies for non-literal sentences, as it is common practice in the psycholinguistic literature.

#### Design and procedure

Testing and experiment were performed in different sessions (on average three sessions for patients, and two for controls) taking place a few days one after the other. The order of testing and experiment was quasi-randomized across participants.

Each experimental trial began with a fixation cross (+) in the center of a computer screen. A spacebar press initiated the presentation of a sentence fragment that was formed by the sentence without the last word (e.g., *Giulia aveva dei grilli per la*). A second spacebar press initiated the target word presentation that could complete the sentence fragment in a sensible or non-sensible way. The target word was written in GENEVA BOLD 14 and appeared in the center of the screen. The presentation of the target word lasted until a response was given. Participants were instructed to press a *YES* button as quickly and accurately as possible when the target word was a good, sensible continuation of the previous sentence fragment (e.g., *TESTA*) and a *NO* button when the target word was a bad, non-sensible continuation (e.g., *SPUGNA*). The positions of the response buttons were counterbalanced across participants. An experimenter sat behind the patient to ensure that s/he was pressing the spacebar for advancing in the sentence presentation and the response buttons for responding (which always happened). Each participant performed 10 practice trials formed by five literal sentences ending with sensible continuations and five with non-sensible continuations. The practice was followed by the 152 experimental trials. Stimulus presentation and response collection were performed using a purpose-written E-Prime script (Psychology Software Tools).

### Statistical analyses

**Figure 1:**
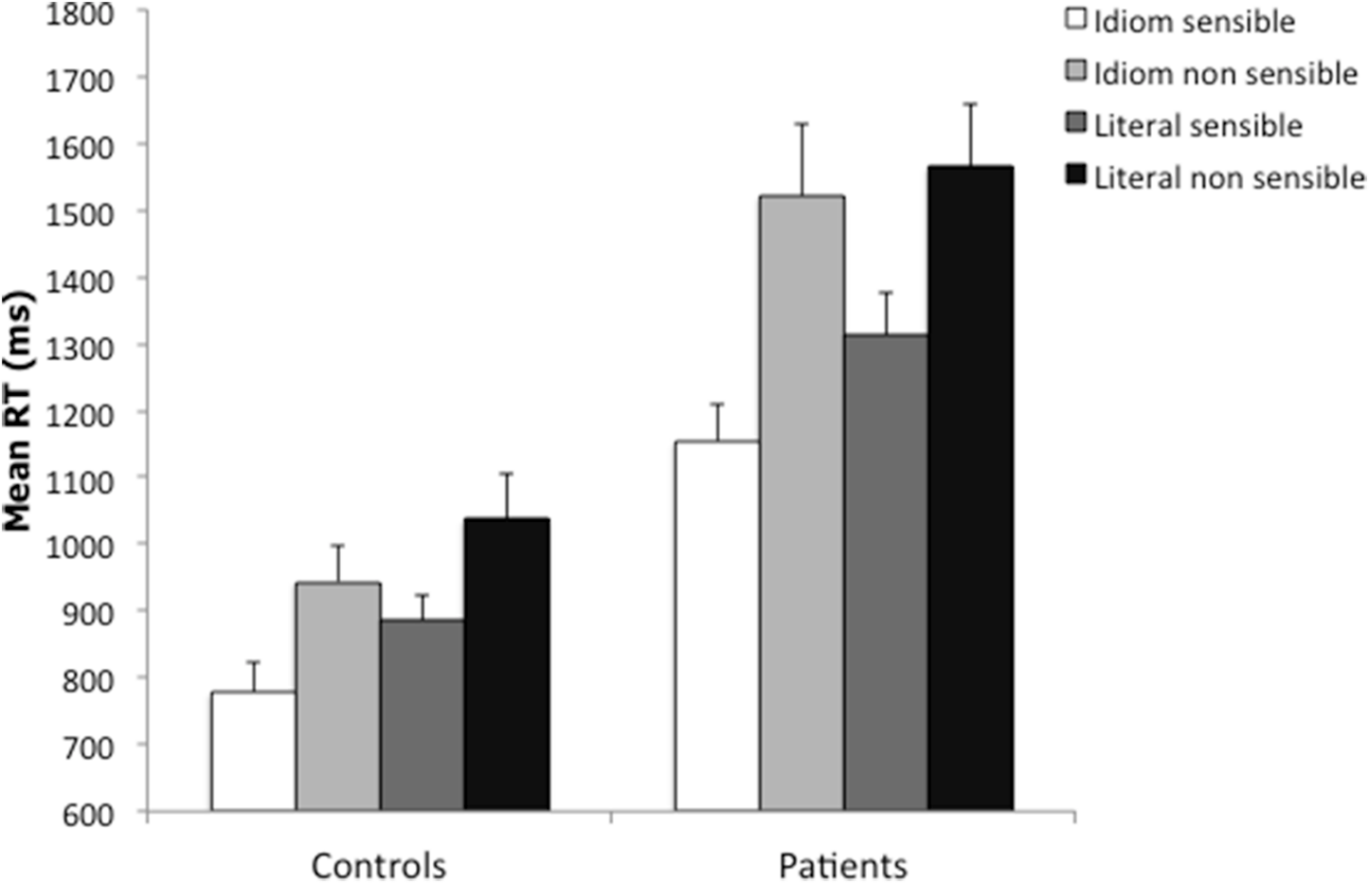
**Mean reaction times for controls and patients in idiom sensible (white bar), idiom non-sensible (bright gray bar), literal sensible (dark gray bar), and literal non-sensible (black bar) sentences**. Error bars represent standard errors of the mean.

**Figure 2:**
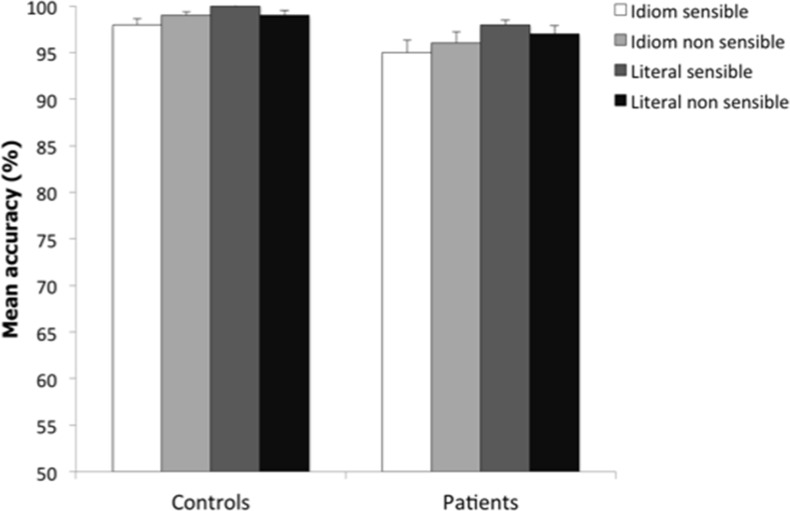
**Mean percentage of correct responses for controls and patients in idiom sensible (white bar), idiom non-sensible (bright gray bar), literal sensible (dark gray bar), and literal non-sensible (black bar) sentences**. Error bars represent standard errors of the mean.

The mean RTs to correct answers and the accuracy proportions of patients and healthy controls in the different conditions are plotted in Figures 1, 2. The mean RTs of correct responses and the accuracy proportions were submitted to analyses of covariance (ANCOVAs) to control for confounding effects accounted for by the following covariates: Verbal fluencies (phonemic and semantic), Vocabulary, and Digit span. Group (patients vs. controls) was a between-subject factor, Sentence (idiomatic vs. literal) and Continuation (sensible vs. non-sensible) within-subject factors. *Post-hoc* Newman-Keuls tests were employed to further examine significant interactions (α = 0.05). Comparing healthy subjects and patients may raise a reliability issue for the effects in an ANCOVA design. Thus, we checked the reliability of significant effects from the ANCOVAs by estimating the sampling distribution under the null-hypothesis that no difference exists between healthy subjects and patients using a non-parametric bootstrap procedure (Efron and Tibshirani, 1993; Di Nocera and Ferlazzo, 2000). Namely, on each step: (1) we re-sampled with replacement from the original set of data creating two bootstrap samples, thus making the null-hypothesis true; and (2) the ANCOVA was performed on the bootstrap samples. The procedure was repeated 10,000 times in order to obtain the empirical F distribution under the null-hypothesis. The empirical distribution was then used to estimate the probability of the original *F*-values under the null-hypothesis. The probability values obtained through the bootstrap procedure are hereafter denoted as *p*_boot_.

The effect of prior context on target words was also operationalized in terms of a priming score (PRI) defined as percentage of facilitation [(RT_unrelatedtargets_ – RT_relatedtargets_)/RT_unrelatedtargets_)^*^100] (Spitzer et al., 1993; Kiefer et al., 2009) in the RTs to correct answers. We calculated the PRI for each participant in each condition and entered it in an ANOVA with Group as a between-subject factor and Sentence as a within-subject factor.

To qualify the nature of our effects determining the specific contributions of cognitive, illness-related, and demographical variables to patients' performance, we computed hierarchical regression analyses on the RTs to correct answers using blockwise entry. Twelve predictor variables divided in three blocks were entered in the following order: Block 1 was formed by variables assessing general cognitive and linguistic skills [Verbal fluencies (phonemic and semantic), Vocabulary, BADA, IQ, Digit span]; Block 2 was formed by illness-related variables (years of illness, medications, and PANSS Total Scale); and Block 3 by demographic variables (age, sex, education).

Finally, to explore any effects of the severity of thought disorders, we correlated the mean RTs, and accuracy proportions to the scores of specific items of PANSS (i.e., P2, *Conceptual disorganization*, and N5, *Difficulty in abstract thinking or Concretism*) and of the Negative and Positive Subscales of PANSS. A conservative significance threshold of 0.01 was used to correct for the large number of correlations.

### Results

After adjustment by the covariates, the ANCOVA on the mean RTs to correct answers showed significant main effects of Group [*F*_(1, 72)_ = 9.98, *p* < 0.002, *p*_boot_ < 0.001, η^2^_*p*_ = 0.12], with patients overall slower than controls (+478 ms), and of Continuation [*F*_(1, 72)_ = 5.22, *p* < 0.03, *p*_boot_ < 0.001, η^2^_*p*_ = 0.07], with non-sensible continuations overall slower than sensible ones (+234 ms). A significant Group by Sentence by Continuation interaction was also obtained [*F*_(1, 72)_ = 4.33, *p* < 0.04, *p*_boot_ = 0.014, η^2^_*p*_ = 0.06] (see Table 3A). *Post-hoc* tests revealed that patients were significantly faster in responding to sensible than to non-sensible continuations in both literal and idiomatic sentences (Idiomatic sentences: −367 ms, *p* < 0.0001; Literal sentences: −253 ms, *p* < 0.0001), and to idiomatic than to literal continuations when they were sensible (−158 ms, *p* < 0.0003) but not when they were non-sensible (−44 ms). Patients were significantly slower than controls in rejecting non-sensible continuations in literal and idiomatic sentences (+529 ms, *p* < 0.01; +578 ms, *p* < 0.005; respectively) and, at trend level, in accepting sensible literal continuations (+427 ms, *p* = 0.06). Patients did not significantly differ from controls in accepting sensible idiomatic completions (*p* = 0.13). Controls were faster on sensible than non-sensible continuations in literal and idiomatic sentences (Idiomatic: −168, *p* < 0.0001; Literal: −154 ms, *p* < 0.001), and faster on idiomatic than on literal continuations when these were sensible (−108 ms, *p* < 0.01) and non-sensible (−93 ms, *p* < 0.02). No significant effects of the covariates emerged [Vocabulary: *F*_(1, 72)_ = 2.67, *p* = 0.11; Digit span: *F* < 1; Phonemic fluency: *F* < 1; Semantic fluency: *F*_(1, 72)_ = 2.62, *p* = 0.11]. However, the high number of covariates introduced in the analysis may have reduced the statistical power by adding random noise to the model. Hence we conducted a further ANCOVA with the same factors as the previous one but dropping the least significant covariate (i.e., phonemic fluency). The results of this ANCOVA (see Table 3B) mirror the results of the previous one with the exception of two covariates that now show close to significance effects, namely Vocabulary (*p* = 0.066), and Semantic Fluency (*p* = 0.051).

**Table 3A:** **Summary of ANCOVA results for Reaction Times and Accuracy for Group, Sentence, and Continuation while controlling for Phonemic and Semantic fluencies, Vocabulary, and Digit span**.

	***df***	***F***	***p*-value**	**Partial η^**2**^**
**REACTION TIMES**				
Group	1,72	9.98	0.00	0.12
Sentence	1,72	0.01	0.91	0.00
Continuation	1,72	5.22	0.03	0.07
Group × sentence	1,72	0.27	0.61	0.00
Group × continuation	1,72	0.56	0.46	0.01
Sentence × continuation	1,72	3.80	0.06	0.05
Group × sentence × continuation	1,72	4.33	0.04	0.06
Phonemic fluency	1,72	0.55	0.46	0.01
Semantic fluency	1,72	2.63	0.11	0.04
Vocabulary	1,72	2.67	0.11	0.04
Digit span	1,72	0.76	0.39	0.01
**ACCURACY**				
Group	1,72	0.59	0.44	0.01
Sentence	1,72	6.63	0.01	0.08
Continuation	1,72	0.04	0.85	0.01
Group × sentence	1,72	0.15	0.70	0.00
Group × continuation	1,72	0.01	0.97	0.00
Sentence × continuation	1,72	3.74	0.06	0.05
Group × sentence × continuation	1,72	0.28	0.60	0.00
Phonemic fluency	1,72	0.04	0.84	0.00
Semantic fluency	1,72	0.00	0.99	0.00
Vocabulary	1,72	10.36	0.00	0.13

**Table 3B:** **Summary of ANCOVA results for Reaction Times for Group, Sentence, and Continuation while controlling for Semantic fluency, Vocabulary, and Digit span**.

	***df***	***F***	***p*-value**	**Partial η^**2**^**
**Reaction Times**				
Group	1,73	12.42	0.00	0.15
Sentence	1,73	0.01	0.93	0.00
Continuation	1,73	5.28	0.02	0.07
Group × sentence	1,73	0.15	0.71	0.00
Group × continuation	1,73	0.66	0.42	0.01
Sentence × continuation	1,73	3.77	0.06	0.05
Group × sentence × continuation	1,73	4.28	0.04	0.06
Semantic fluency	1,73	3.93	0.05	0.05
Vocabulary	1,73	3.48	0.11	0.05
Digit span	1,73	1.21	0.27	0.02

Since, as we mentioned, slowing of RTs may inflate contextual effects and group differences, we compared the priming scores (PRI) of controls and patients in the different experimental conditions (see Methods Section). The ANOVA revealed only a significant main effect of Sentence [*F*_(1, 76)_ = 4.176, *p* < 0.04, η^2^_*p*_ = 0.052] with higher priming scores in idiomatic than in literal sentences (17.4 vs. 12.2%, respectively). There were suggestive, although statistically indistinguishable, slightly higher percentages of facilitation in patients than in controls especially in idiomatic sentences (idiomatic sentences: 18.6 vs.16.2%, literal sentences: 12.8 vs. 11.6%, respectively for patients and controls).

Significant effects in the hierarchical regression analyses on patients' RTs revealed that Cognitive variables [i.e., Verbal fluencies (phonemic and semantic), Vocabulary, BADA, IQ, Digit span] accounted for 49.4% of the variance [*F*_(6, 32)_ = 5.21, *p* < 0.001, *r*^2^ = 0.49] in the responses to sensible idiomatic continuations [*F*_(12, 26)_ = 3.14, *p* < 0.007, *r*^2^ = 0.59] with significant contributions of Digit span and IQ [*t*_(32)_ = −2.04, *p* < 0.05; *t*_(32)_ = 2.12, *p* < 0.04, respectively]. None of the blocks produced significant *r*^2^ changes in sensible literal continuations [*F*_(12, 26)_ = 2.18, *p* < 0.05, *r*^2^ = 0.47]. Cognitive variables also accounted for 34.5% of the variance [*F*_(6, 32)_ = 2.81, *p* < 0.03, *r*^2^ = 0.34] in non-sensible literal continuations [*F*_(12, 26)_ = 2.16, *p* < 0.05, *r*^2^ = 0.5] with a significant contribution of Digit span [*t*_(32)_ = −2.4, *p* < 0.02].

The ANCOVA on accuracy^4^ (see Table 3A) revealed a significant main effect of Sentence [*F*_(1, 72)_ = 6.63, *p* < 0.01, *p*_boot_ < 0.002, η^2^_*p*_ = 0.08] with higher accuracy in literal than in idiomatic sentences (98.5 and 97%, respectively). The only covariate leading to a statistically reliable effect was Vocabulary [*F*_(1, 72)_ = 10.36, *p* < 0.001, η^2^_*p*_ = 0.13].

### Effects of clinical variables

The correlations of the scores in items P2 and N5 of PANSS^5^ and RTs and accuracy did not yield any significant results (α = 0.01). However, some results significant at trend level merit reporting. Specifically, *Conceptual disorganization scores* (P2) correlated positively with the RTs to sensible and non-sensible idiomatic continuations (*p* = 0.04; *p* = 0.05, respectively), and inversely with accuracy in responding to non-sensible idiomatic and literal continuations (*p* = 0.02; *p* = 0.02, respectively). Then, again at trend level, *Difficulty in abstract thought or Concretism* scores (N5) correlated positively with the RTs to sensible idiomatic continuations (*p* = 0.02), and inversely with accuracy in non-sensible literal continuations (*p* = 0.03). The Negative scale scores positively correlated with the RTs to sensible idiomatic continuations (*p* = 0.009), and to sensible and non-sensible literal continuations (but at trend levels: *p* = 0.02; *p* = 0.04, respectively). Accuracy in responding to non-sensible idiomatic and literal continuations inversely correlated with Negative scale scores (*p* = 0.007; *p* = 0.004, respectively) and with Positive scale scores (*p* = 0.01; *p* = 0.01, respectively).

## Discussion and conclusions

In normal sentence processing, comprehenders constantly compute the relationships between individual words in a combinatorial way and compare this information with the relationships that are prestored within semantic memory (Kuperberg, 2007, 2010a,b). Semantic memory-based stream of analysis occurs partly in parallel with the combinatorial stream of analysis in which the lexical-semantic information of individual words is integrated compositionally with morphosyntactic and thematic structures to determine the sentence meaning. It has been proposed (Kuperberg, 2007, 2010a,b) that in SZ patients imbalance between the two streams of analysis may lead to sentence comprehension deficit due to over-reliance of semantic-memory based activity at the expense of the combinatorial integrative stream of analysis. Inspired by the *Dual Stream hypothesis* of Kuperberg (2010b), we explored the possibility that idiom comprehension may be relatively spared in SZ patients when idioms are familiar, literally implausible, and predictable before offset. Idiomatic meanings should in fact be directly retrieved from semantic memory; hence patients' over-reliance on a semantic memory-based stream of analysis may turn into a processing resource rather than a limitation. Paradoxically, and despite equally high predictability of sentence-final words, patients' performance may be poorer in literal sentence that instead require syntactic and semantic integration of the constituent word meanings. This may lead to a patients' performance close to controls in idiomatic but not in literal sentences.

Our results showed that patients were overall slower than healthy controls (+478 ms), as expected given the documented general slowing down of SZ patients. Patients were faster in correctly responding to sensible than to non-sensible continuations in both idiomatic and literal sentences. They also were faster in responding to sensible idiomatic continuations than to sensible literal ones, in line with our hypothesis of an advantage driven by the conventionalized nature of idioms. The ANCOVA and the regression analyses showed that cognitive variables indeed played a role in shaping the comprehension performance of patients in line with the evidence of a generalized intellectual impairment of SZ patients even when, as the patients tested in this study, they were relatively well-functioning. Once the contribution of the covariates was partialled out, results showed that patients were slower than controls in correctly rejecting non-sensible literal and idiomatic sentences, and in accepting sensible literal continuations. The RTs of patients to idiomatic sentences were still slower than those of controls but this difference was not statistically significant. This cannot be taken to imply that patients comprehended idioms as controls. Rather these results showed that the state of residual schizophrenia did not contribute to slower processing of sensible idioms above and beyond the cognitive deficits that characterized patients. This was clarified by the results of the hierarchical regression analysis that showed that the reaction times to sensible idioms (and to literal non-sensible sentences) were compellingly explained by differences in the cognitive variables (notably, verbal memory and IQ for sensible idioms, and verbal memory for non-sensible literal sentences). In sum the cognitive dysfunction of the SZ patients tested in this study affected the comprehension of idiomatic as well as of literal sentences, and it was even more pronounced for literal, compositional sentences, in line with our predictions. It should be noted that we measured reaction times to the sentence-final words which may differ from the processing of words within a sentence. In fact, wrap-up effects at the end of sentences place the highest demands on literal, combinatorial processing (Kuperberg et al., 2010).

Patients' accuracy was close to that of controls (96.5 vs. 98.5%, respectively), in contrast to prior studies (e.g., Iakimova et al., 2005, 2010; Thoma et al., 2009; Schettino et al., 2010). We cannot exclude that the lack of a group difference on accuracy across the different experimental conditions may reflect a ceiling effect. Scores in the Vocabulary subtest of WAIS had a general effect on accuracy, a result of interest given that this subtest of WAIS is believed to tap premorbid intelligence in SZ (Lezak et al., 2004) and the documented association of verbal intelligence to efficient sentence comprehension (Hunt, 1977).

The analyses of the priming scores (PRIs) revealed a stronger effect of idiomatic than of literal contexts on target words. It is unlikely that this effect may be due to predictability since sentence-final words were equally highly predictable in both types of sentence. Rather, it seems to reflect the conventionalized, bound nature of idiom strings. In fact, when overlearned figurative expressions are familiar *they provide a degree of context and cloze probability significantly beyond that of literal statements* (Strandburg et al., 1997, p. 605).

In sum, idiom-final words seemed to be more accessible^6^ to SZ patients than literal-final words, but the processing of both types of words was severely affected by the patients' cognitive abnormalities. Regression analysis showed that cognitive variables (notably, verbal memory and IQ) accounted for a high amount of variance in patients' RTs to sensible idioms and to non-sensible literal sentences. Specifically, short-term verbal memory had a specific role on RTs to non-sensible literal sentences, and both short-term verbal memory and IQ^7^ on sensible idioms. Prior studies reported mixed evidence on the effects of patients' IQ: it affected idiom comprehension in Iakimova et al. (2010) but was not a significant predictor of correct responses to idioms in Schettino et al. (2010). In Varga et al. (2014) SZ patients with lower IQ were impaired in comprehending unconventional metaphors and irony while performing close to controls in comprehending conventional metaphors (that could in principle be similar to idioms, although no examples are provided in the study). Higher IQ patients performed overall as well as controls. A previous study by Kazmersky et al. (2003) also reported evidence of a link between IQ and figurative language comprehension in healthy participants in that individuals with lower IQ had more difficulty in understanding figurative language than higher IQ individuals.

Correlations showed some effects of the severity of thought disorder on patients' performance, although of limited nature given the clinical profile of patients. In fact RTs tend to slow down as *Conceptual disorganization*, *Difficulty in abstract thinking*, and negative symptoms increased within the patients group. Specifically, higher scores in the item *Conceptual disorganization* (P2) of PANSS were associated with longer RTs to idioms and decreased accuracy on non-sensible sentences (no matter whether literal or idiomatic). This is consistent with evidence that high scores in P2 reflect semantic processing dysfunction (Kiefer et al., 2009), We also found that higher scores in *Difficulty in abstract thinking or Concretism* (N5) led to longer RTs to sensible idioms (as in Iakimova et al., 2010) and decreased accuracy on non-sensible literal sentences. N5 scores are thought to reflect deficient comprehension of abstract, non-literal language (e.g., Kircher et al., 2007; Iakimova et al., 2010; Mashal et al., 2013), as confirmed by recent brain imaging evidence (Kircher et al., 2007; Mashal et al., 2013) that reduced brain activation (in the left IFG and left MFG) during non-literal language comprehension was correlated to high scores in N5. Higher scores in the Negative Scale of PANSS led to longer RTs to sensible idiomatic continuations and to literal ones (sensible and non-sensible). This would be consistent with the claim that severity of negative symptoms is associated with deficits in executive functions (e.g., Basso et al., 1998; O'Leary et al., 2000; Schettino et al., 2010) that brain-imaging studies (e.g., Zempleni et al., 2007; Romero Lauro et al., 2008; Proverbio et al., 2009) showed to be relevant to language comprehension, and particularly to idiom comprehension. Lastly, higher scores in the Positive Scale, as well as in the Negative Scale, were associated with decreased accuracy in rejecting non-sensible literal and idiomatic continuations. This would conform to evidence that increase in the severity of positive symptoms is linked to meaning processing deficits (Kuperberg and Heckers, 2000; Brüne and Bodenstein, 2005; Salisbury, 2008; Iakimova et al., 2010). Overall, these results indicate that language comprehension in patients with more severe psychopathology was defective in several respects that included differentiating between idiomatic and semantically incongruous literal sentences. This suggests that the ability to comprehend idiomatic expressions and to differentiate conventionalized from anomalous expressions may be indicative of the severity of the linguistic and cognitive deficits of SZ patients. Improving this ability may also constitute a promising path for the treatment of cognitive deficits in SZ patients. In sum, in line with prior evidence (Ditman and Kuperberg, 2007; Titone et al., 2007), our results suggest that even though SZ did not necessarily bring to a loss of semantic-lexical knowledge, definitively it modifies the mechanisms whereby this knowledge is retrieved.

There are some limitations to our study that need to be addressed. First, inclusion criteria may have resulted in a patient sample with mild-to-moderate average levels of psychopathology and this may have limited the potential for detecting possible correlations with clinical variables due to floor effects. Second, patients were tested while they were clinically stabilized hence limiting any conclusions on the exact nature of the language processing perturbations in paranoid SZ. Third, patients were on antipsychotic medication (mostly second-generation antipsychotic medication); hence an effect of treatment could not be ruled out. Fourth, patients and controls were matched in education. Controlling for a factor as education that may account for some variance in neuropsychological measures may remove variance attributable to the variable of interest. Lastly, we only tested patients with paranoid SZ without any comparisons with other forms of SZ. Whatever the case, our results would still be relevant insofar as they show that there is not a global language dysfunction in mild-to-moderate paranoid SZ but qualitatively different language processing impairments that differently affect literal and non-literal language. This may shed some further light on the complexity of the neural underpinnings of literal and non-literal language comprehension as well as on the manifestations of this neurodevelopmental disorder.

As we mentioned in the Introduction, the neural correlates of SZ partly overlap with the functional neuroanatomy of idioms. In fact, converging evidence on language-impaired and language-unimpaired subjects coming from lesion studies, rTMs, and fMRI studies (for overviews, see Thoma and Daum, 2006; Bohrn et al., 2012; Cacciari and Papagno, 2012; Rapp et al., 2012) showed that idiom comprehension is based on a complex neural network that includes the temporal cortex, the superior medial frontal gyrus and the inferior frontal gyrus in the left hemisphere; and the superior and middle temporal gyri, the temporal pole and the inferior frontal gyrus in the right hemisphere, with more extended activations in the left than in the right hemisphere. This neural architecture is not solely involved in idiom comprehension. For instance, idioms and metaphors have largely overlapping activation foci in the left hemisphere (e.g., in the left inferior frontal gyrus) together with important differences concerning a more extended activation in the dorso-lateral prefrontal cortex for idioms than for metaphors, and different clusters of activation in the right inferior frontal gyrus (Bohrn et al., 2012) and right middle temporal gyrus (Rapp et al., 2012) for metaphors than for idioms that may in part depend on the novelty of metaphorical meanings. To the best of our knowledge, so far none of the studies on figurative language comprehension in SZ tested the comprehension of idioms and metaphors within the same sample of patients. Comparing the comprehension of conventional, prestored idiomatic meanings to that of novel, unconventional metaphors would instead provide important evidence on the neural underpinnings of non-literal language comprehension and on whether SZ patients may indeed be favored by the prefabricated nature of idioms as compared to the computation of novel metaphorical meanings that require the blending of distant semantic domains.

## Author contributions

Cristina Cacciari initially conceived the idea for the study which was then further developed and finalized by Cristina Cacciari, Francesca Pesciarelli, and Tania Gamberoni. The stimulus materials were prepared by Cristina Cacciari with the help of a doctoral student. Data collection was made possible by Tania Gamberoni, Leo Lo Russo, Francesca Pedrazzi, Ermanno Melati, and Francesca Pesciarelli. Analyses were run by Fabio Ferlazzo and Francesca Pesciarelli. The majority of this paper was written by Cristina Cacciari and Francesca Pesciarelli.

### Conflict of interest statement

The authors declare that the research was conducted in the absence of any commercial or financial relationships that could be construed as a potential conflict of interest.

## Footnotes

^1^Semantic priming occurs whenever there is more efficient processing of a target word when preceded by a related stimulus or context.

^2^Only two studies reported idiom predictability scores (low scores in Titone et al., 2002 and medium scores in Iakimova et al., 2010).

^3^According to PANSS classification criteria, Total scores up to 58 are indicative of a mild form of psychopathology, and up to 75 of a moderate form.

^4^A qualitative analysis of the patients' errors revealed that they made a slightly higher number of errors in rejecting non-sensible idiomatic continuations than in accepting sensible idiomatic ones (35 vs. 28, respectively), and a slightly higher number of errors in accepting sensible literal continuations than in rejecting non-sensible literal ones (22 vs. 15, respectively).

^5^According to PANSS criteria, in our patient sample the severity of Conceptual disorganization (P2) went from absent to moderate (mean score = 1.67, range = 1–4, *SD* = 0.91) and Difficulty in abstract thought or Concretism (N5) from absent to mild (mean score = 2.00, range = 1–3, *SD* = 0.79). The low average scores in these two items, and in general in the Negative and Positive scales, may have limited the potential for detecting correlations of RTs and accuracy with clinical variables.

^6^Accessibility refers to readiness with which a word is retrieved from semantic memory.

^7^This is consistent with what Verguts and De Boeck(2002; Hunt, 1977) defined as the ubiquitous finding of a substantial correlation between memory capacity and general (fluid) intelligence.
