# Supplementary material for: Is the comprehension of idiomatic sentences indeed impaired in paranoid Schizophrenia? A window into semantic processing deficits
Source: Front Hum Neurosci. 2014 Oct 9;8:799. doi: 10.3389/fnhum.2014.00799 (PMC4190991; doi:10.3389/fnhum.2014.00799)
Supplement: Supplementary file 1 [file DataSheet1.DOCX]

Appendix

Italian idioms with word-by-word and meaning English translations

| Essere il fanalino di coda- To be the taillight |
| --- |
| Essere nel fiore degli anni- To be in the flower of the years, to be in the best years |
| Prendere lucciole per lanterne- To take fireflies for lamps, do not tell chalk from cheese |
| Essere in un mare di guai- To be in a sea of troubles, to be in serious troubles |
| Fare castelli in aria- To build castles in the air |
| Avere un diavolo per capello- To have a devil for hair, to be extremely angry |
| Fare di ogni erba un fascio- To make of each herb a bundle, to lump everything together |
| Sentirsi un tuffo al cuore- To feel a plunge in the heart, to miss a beat of the heart |
| Cavarsela per il rotto della cuffia- To escape from a hole in the cap; to get out by the skin of one’s teeth |
| Saltare di palo infrasca- To jump from pole to branch, to jump from one thing to another |
| Non cavare un ragno dal buco-To not get out a spider from the hole, to go nowhere |
| Avere un lampo di genio- To have a stroke of genius |
| Perdere il filo del discorso- to lose the thread of discourse, of one’s argument |
| Andarsene con la coda tra le gambe- To go away with the tail between the legs, depressed |
| Sentirsi in una botte di ferro- To feel in an iron barrel, to be impregnable |
| Dare il colpo di grazia- To give the blow of grace, to give a finishing stroke |
| Mettere il carro davanti ai buoi- To put the chart before the oxen, horse |
| Sputare nel piatto in cui si mangia- To spit in the dish where you eat, to be ungrateful |
| Far venire il latte alle ginocchia- To make the milk come to the knees, to be boring |
| Prendere due piccioni con una fava- To take two pigeons with one broad bean, kill two birds with one stone |
| Fare orecchie da mercante- to make the ears of a merchant, to turn a deaf ear |
| Mettersi il cuore in pace –To put the heart at rest |
| Navigare in cattive acque– To navigate in bad waters, in deep water |
| Mettere la testa a posto- To put the head in site, to settle down |
| Levarsi un peso dallo stomaco- To get off a weight from the stomach, to take a great weight off one’s mind |
| Finire in una bolla di sapone- To end in a soap bubble, in nothing |
| Non essere farina del suo sacco-To not be flour of one’s own sack, someone else had a finger in this pie |
| Tirare l’acqua al proprio mulino- To pull water to one’s own mill– to pursue one’s own interest |
| Avere dei grilli per la testa- To have some crickets for the hair, to be full of strange ideas |
| Perdere il lume della ragione- To lose the light of reason, to lose one’s temper |
| Non avere peli sulla lingua- To not have hair on the tongue, to be very outspoken |
| Toccare il cielo con un dito- To touch the sky with a finger, to be extremely happy |
| Metterci una croce sopra- To put a cross over, to renounce |
| Piangere sul latte versato-To cry over spilt milk |
| Avere la testa fra le nuvole- To have the head among the clouds, to be absent-minded |
| Camminare sul filo del rasoio- To walk on the hedge of the razor |
| Avere la puzza sotto il naso- To have the stink under the nose, to be standoffish |
| Cogliere con le mani nel sacco- To catch with the hands in the sack, to catch red-handed |
